# Scoping review to identify potential non-antimicrobial interventions to mitigate antimicrobial resistance in commensal enteric bacteria in North American cattle production systems

**DOI:** 10.1017/S0950268815000722

**Published:** 2015-04-23

**Authors:** C. P. MURPHY, V. R. FAJT, H. M. SCOTT, M. J. FOSTER, P. WICKWIRE, S. A. McEWEN

**Affiliations:** 1 Population Medicine, University of Guelph, Guelph, Ontario, Canada; 2 Department of Veterinary Physiology and Pharmacology, Texas A&M University, TX, USA; 3 Department of Veterinary Pathobiology, Texas A&M University, TX, USA; 4 Medical Sciences Library, Texas A&M University, TX, USA; 5 College of Medicine, University of Illinois, IL, USA

**Keywords:** Antibiotic resistance, antimicrobial resistance in agricultural settings, epidemiology, veterinary epidemiology

## Abstract

A scoping review was conducted to identify modifiable non-antimicrobial factors to reduce the occurrence of antimicrobial resistance in cattle populations. Searches were developed to retrieve peer-reviewed published studies in animal, human and *in vitro* microbial populations. Citations were retained when modifiable non-antimicrobial factors or interventions potentially associated with antimicrobial resistance were described. Studies described resistance in five bacterial genera, species or types, and 40 antimicrobials. Modifiable non-antimicrobial factors or interventions ranged widely in type, and the depth of evidence in animal populations was shallow. Specific associations between a factor or intervention with antimicrobial resistance in a population (e.g. associations between organic systems and tetracycline susceptibility in *E. coli* from cattle) were reported in a maximum of three studies. The identified non-antimicrobial factors or interventions were classified into 16 themes. Most reported associations between the non-antimicrobial modifiable factors or interventions and antimicrobial resistance were not statistically significant (*P* > 0·05 and a confidence interval including 1), but when significant, the results were not consistent in direction (increase or decrease in antimicrobial resistance) or magnitude. Research is needed to better understand the impacts of promising modifiable factors or interventions on the occurrence of antimicrobial resistance before any recommendations can be offered or adopted.

## INTRODUCTION

Shortly after the discovery of antimicrobials and their introduction into clinical practice, acquired antimicrobial resistance was observed to adversely impact clinical outcomes [1]. As antimicrobial resistance threatens the efficacy of antimicrobial treatment for bacterial infections in any animal species, it is a topic of much research in the human, veterinary, agri-food and environmental sectors. The epidemiology of antimicrobial resistance is complex and involves links between humans, animals and the environment, including the transmission of antimicrobial-resistant bacteria within and between these niches. Public health concerns about the transmission of antimicrobial resistant foodborne pathogens or genes through the food chain have existed for decades. These concerns continue with the isolation of extended-spectrum cephalosporin- [2] and carbapenem-resistant [2–4] *E. coli* and *Salmonella enterica* in livestock.

Human acquisition of bacteria carrying antimicrobial resistance genes from cattle and other farmed animals may occur by direct animal contact, via the food chain, or through contamination of the environment. Consumption of beef products has been linked to foodborne outbreaks of infection including those caused by antimicrobial-resistant bacteria [5]. Infection with antimicrobial-resistant organisms has been associated with more adverse health outcomes compared to infection with pan-susceptible strains. For example, patients with antimicrobial resistant *Salmonella* were at greater risk of hospitalization, increased duration of hospitalization and bloodstream infections [5]. Because of the adverse human health outcomes and the emergence of resistance in animals and food to antimicrobials of notable public health importance [2, 4], determining modifiable factors or interventions to reduce the occurrence of antimicrobial resistance in cattle populations is desirable.

Antimicrobial resistance is a broad, widely researched topic with a large volume of peer-review published research. Links between antimicrobial uses in agriculture with antimicrobial resistance in human health have been documented [6–14]. Consequently, some interventions that focus on reduction of antimicrobial use have been applied in certain agricultural settings to mitigate the transfer of antimicrobial resistant bacteria or determinants along the food chain [11, 15]. However, modifiable non-antimicrobial factors or interventions may also contribute to a reduction in the transfer of resistant bacteria or determinants and a reduction in the occurrence of antimicrobial resistance. Modifiable non-antimicrobial factors are factors other than antimicrobial use or exposure that may be changed to alter the occurrence of antimicrobial resistance such as type of production system (conventional or non-conventional), stocking density and hygiene. The relationships of non-antimicrobial factors to antimicrobial resistance may be direct, but likely also have relationships with illness, antimicrobial treatment and use, other routes of antimicrobial exposure and each other. Modifying non-antimicrobial factors may impact the occurrence of antimicrobial resistance through more than one causal pathway. A structured synthesis of published research is required to more fully understand the current state of knowledge, to identify plausible and practical non-antimicrobial interventions, and to identify and prioritize research needs and knowledge gaps.

The scoping review is a newer method of review with over 70% of scoping reviews in the health sector published since 2010 [16]; notably, they have also been employed in the agri-food research arena [17–19]. Scoping review methodologies are appropriate for the review of broad topics [20] and they can be used to map the distribution and characteristics of a particular topic or issue, summarize the state of knowledge, identify research gaps and inform future research, provide data to stakeholders, and help prioritize questions for more focused systematic review [20]. Our research question was: What modifiable non-antimicrobial factors or interventions have the potential to decrease the prevalence of organisms with phenotypic or genotypic expression of resistance, to decrease the populations of organisms carrying resistance genes, or to prevent the accumulation of resistance (i.e. reduce selection pressure for resistance) in North American cattle production systems? The scoping review approach was a better fit to our broad research question than the more focused systematic review method. We searched for, and reviewed, published peer-reviewed English-language literature (without geographical limitations) covering animal populations with additional data arising from human populations and *in vitro* studies to identify, characterize, and summarize potential modifiable non-antimicrobial interventions in order to reduce the occurrence of antimicrobial resistance among enteric bacteria in North American cattle populations.

## MATERIALS AND METHODS

### Search terms and strategy

**Fig. 1.**
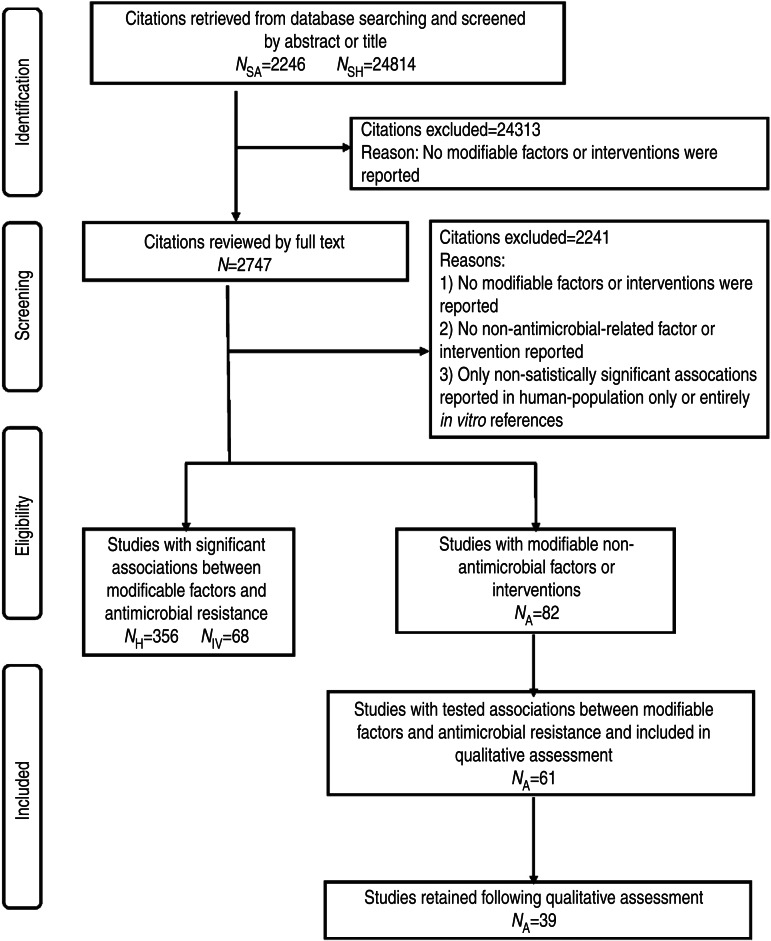
PRISMA flow chart documenting the literature retrieval* and inclusion/exclusion criteria for citations to identify modifiable non-antimicrobial factors or interventions to reduce the occurrence of antimicrobial resistance in cattle production systems. (* *N*_SA_, Search designed to return citations studying animal populations. *N*_SH_, Search designed to return citations studying human or *in vitro* populations. *N*_H_, Citations studying human-only populations. *N*_IV_, Citations studying entirely *in vitro* populations. *N*_A_, Citations studying animal populations.)

Using the research question stated above, searches were developed to return citations of investigations into non-antimicrobial factors associated with antimicrobial resistance in animal populations, human populations and *in vitro* (Fig. 1). For animal population citations, the search focused on antimicrobial resistance in enteric or faecal bacteria and used multiple broad and specific search terms for antimicrobial susceptibility and animal population (Supplementary Appendix 1, available online). This search used eight databases in the OVID platform (Medline, EMBASE, CAB abstracts, Biosis, Zoological Records, Agris, Global Health, Food Science) as well as three databases through the Proquest, formerly Cambridge Scientific Abstracts (CSA), interface (Agricola, Biological Sciences, Toxline) and was performed from 12 June to 13 July 2011. The search for citations of studies conducted in human populations or *in vitro* included general and specific terms for bacteria (e.g. Enterobacteriaceae or *Enterococcus* or *Escherichia coli*), general antimicrobial susceptibility terms, terms related to changing antimicrobial susceptibilities (e.g. affect or effect or reduce or decrease), and factors or interventions related to particular actions (e.g. infection control) or pharmacology (e.g. dose-response relationship, pharmacodynamics) (Supplementary Appendix 2). Medline and CAB Abstracts were searched from 13 October to 31 December 2011. All searches included all available years of the databases, all geographical locations and were limited to those published in English. All citations were exported and de-duplicated (electronically and manually) in a web-based bibliographical database manager (RefWorks 2.0; http://www.refworks.com/).

### Relevance screening of abstracts and full-text citation

Each abstract (or title only, where no abstract was available) was screened using the question: ‘Does this citation describe modifiable non-antimicrobial interventions that may change antimicrobial resistance, or else modifiable factors that may be associated with antimicrobial resistance’? Citations reporting modifiable factors or interventions were retained, and citations reporting only non-modifiable factors such as breed, sex, or geographical location were excluded. Full text citations were screened with the same question as the abstracts. All screening of abstracts and review of full text citation was performed independently by two reviewers. The review team included three veterinary epidemiologists with expertise in the subject matter, a veterinary pharmacologist and a research assistant. Citations were included in the review or excluded with complete agreement by the two reviewers. In the case of disagreement, the citation was included or excluded on the basis of a review by a third member of the team.

### Data extraction from citations of studies investigating non-antimicrobial use interventions or factors

Data on the following were extracted from full-text citations of studies conducted in animal populations (including studies also involving a human population): animal species, study design, reported modifiable non-antimicrobial risk factors and interventions, referent or control group, bacteria or bacterial genes isolated, antimicrobial susceptibility or antimicrobial resistance genes, the association(s) between antimicrobial susceptibility and non-modifiable risk factors (i.e. significant, non-significant), and the direction of the associations. Multiple drug resistance was extracted from those studies where multiple drug resistance was defined and investigated, and from studies where susceptibility to two or more antimicrobials in combination was reported (excluding the combinations of trimethoprim-sulfamethoxazole and quinpristin-dalfopristin).

For exclusively human or *in vitro* studies, extracted data were limited to study population, study design, modifiable factor or intervention, and the direction of the associations. We placed this limitation because data from studies conducted in animal populations were assumed to be of greater relevance to our objective, and the much larger volume of citations in human populations exceeded our capacity for full data extraction. Thus, resources were mainly allocated to detailed data extraction from animal population studies and their qualitative review (see below).

For all studies, specific modifiable non-antimicrobial factors and interventions were aggregated into categories. The categories were not established *a priori*, but were developed iteratively by two independent reviewers. Separate categories were developed for studies in animal, human and *in vitro* populations, and the categories were then reviewed and evaluated qualitatively for common themes.

### Quality assessment of animal-population studies

An assessment was made of the quality of studies in animal populations only. Any studies reporting only descriptive or raw data were excluded from the assessment. The purpose of the assessment was to identify and remove from the review any studies with methodological flaws or biases that would adversely affect the validity of the scoping review. This quality assessment instrument was a pretested questionnaire that was adapted from the GRADE assessment and risk-of-bias approach [21] (Supplementary Appendix 3). Two reviewers independently evaluated the stated inclusion/exclusion criteria for observational studies, or group allocation for randomized or experimental studies, assessed the impact of randomization (or lack of randomization) where appropriate, evaluated appropriateness of the statistical analysis, reviewed whether other procedures were defined, appropriate and used correctly, and determined if biases or uncontrolled confounders may have influenced the validity of the results. Studies were ranked either as high, moderate, or low quality, or else unreliable. In cases of disagreement, a third person reviewed the study, which was then classified on the basis of the third review. Only those studies ranked as being of high or moderate quality were retained.

## RESULTS

**Fig. 2.**
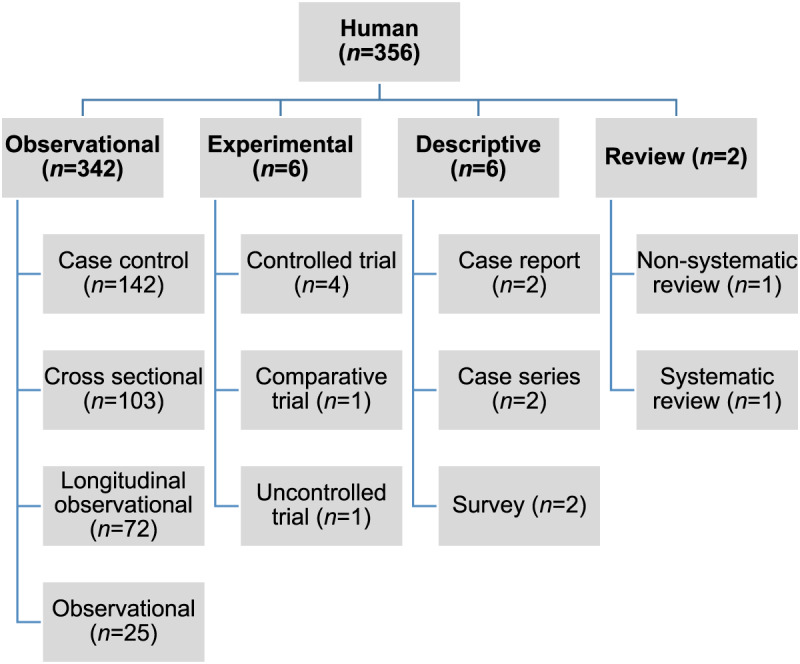
Numbers of citations by study type reporting statistically significant results between non-antimicrobial factors and antimicrobial resistance in human populations.

**Fig. 3.**
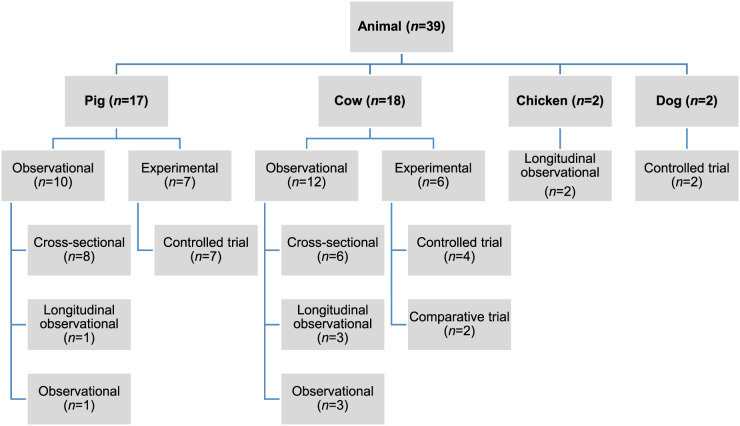
Distribution of study design from retained citations studying non-antimicrobial factors associated with antimicrobial resistance in animal populations.

Of the 27 060 de-duplicated citations, 506 reported on modifiable non-antimicrobial factors or interventions. Most of these described studies in human populations exclusively (*n* = 356, 70%), 16% (*n* = 82) involved animal populations, and 13% (*n* = 68) were conducted entirely *in vitro* (Fig. 1). Of the retained citations involving human and animal populations, the most common study type was observational (*n* = 366, 93%) (Figs 2 and 3).

**Fig. 4.**
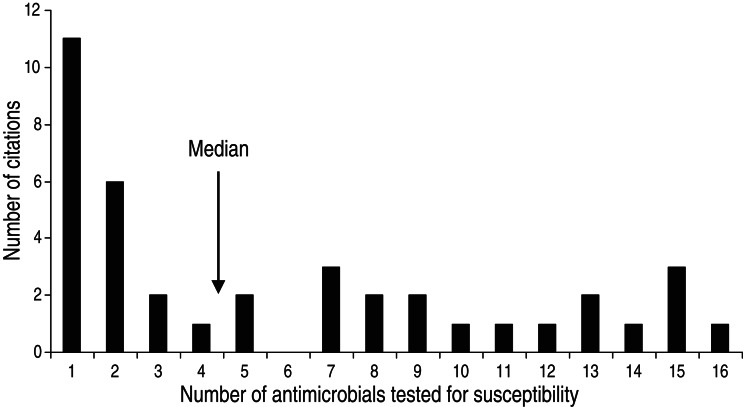
Number of antimicrobials* tested for susceptibility in in animal population citations reporting associations between non-antimicrobial factors or interventions and antimicrobial resistance. [* Including ‘any resistance’ (as defined by the authors) or multiple drug resistance (as defined by the authors) or any two or more antimicrobial combinations reported (excluding trimethoprim-sulfamethoxazole and quinpristin-dalfopristin).]

The animal populations studied in the retained studies after the qualitative assessment (*n* = 39) were cattle (*n* = 18), pigs (*n* = 17), chickens (*n* = 2) and dogs (*n* = 2). There were five bacterial genera, species or types investigated individually or in combination: *E. coli* (*n* = 21, 54%), *Salmonella enterica* (*n* = 8, 20%), *Campylobacter* species (*n* = 7, 18%), coliforms (*n* = 5, 13%), and *Enterococcus* species (*n* = 3, 8%). Two studies investigated antimicrobial resistance genes. Most studies examined only one bacterial genus or species (*n* = 31, 79%), 15% (*n* = 6) studied two and 5% (*n* = 2) studied three. Overall, antimicrobial susceptibility to 40 different antimicrobials from 14 antimicrobial classes was reported (Table 1), and the median number of antimicrobials included in a study was three (range 1–16) (Fig. 4). There were 485 associations between specific modifiable factors or interventions and antimicrobial susceptibility (or genes in a specific bacterial species) reported, and specific bacteria/antimicrobial susceptibility combinations (e.g. *E. coli* and tetracycline) occurred at a frequency of ⩽5%. The most frequent combinations were *E. coli* and tetracycline, ampicillin, apramycin, gentamicin, or streptomycin, and *Campylobacter* species and ciprofloxacin (Table 2).

**Table 1.** Descriptions of non-antimicrobial factors, tested antimicrobials, bacteria and, animal species in animal population citations (n = 39) that investigated associations between antimicrobial resistance and non-antimicrobial factors

Antimicrobial class	Antimicrobial (n*)	Bacteria (n*)	Animal species (n*)	Description of factor(s)	Reference
Aminoglycosides	Apramycin (*n* = 5)	*E. coli* (*n* = 5), *Salmonella enterica* (*n* = 1)	Pig (*n* = 4), cow (*n* = 1)	Non-conventional management†; Contact with other animals treated with antimicrobials; Treatment with oral competitive exclusion culture made from piglets	[28–32]
	Gentamicin (*n* = 12)	*E. coli* (*n* = 6), *Enterococcus* species (*n* = 1), *Campylobacter* species (*n* = 3)	Cow (*n* = 5), pig (*n* = 5), chicken (*n* = 2)	Non-conventional management; Feeding silage or grain; Dietary zinc or copper	[32–43]
	Kanamycin (*n* = 9)	*E. coli* (*n* = 4), *Campylobacter* species (*n* = 1), *Salmonella enteria* (*n* = 3), coliforms (*n* = 2)	Pig (*n* = 4), cow (*n* = 3), chicken (*n* = 2)	Non-conventional management	[32, 33, 35, 37, 39, 40, 43–45]
	Neomycin (*n* = 4)	*E. coli* (*n* = 2), coliforms (*n* = 2)	Cow (*n* = 1), pig (*n* = 3)	Non-conventional management	[44–47]
	Spectinomycin (*n* = 1)	*Enterococcus* species (*n* = 1)	Pig (*n* = 1)	Herd size; Bringing new animals onto the farm	[36]
	Streptomycin (*n* = 14)	*E. coli* (*n* = 7), *Campylobacter* species (*n* = 2), *Salmonella enterica* (*n* = 2)	Cow (*n* = 5), pig (*n* = 7), chicken (*n* = 2)	Non-conventional management; Health status; Treatment with oral competitive exclusion culture made from piglets; Dietary zinc or copper; Pen/group/herd size; Number of suppliers; Education; Bringing new animals onto the farm	[32, 33, 35, 37–39, 44–49]
	Vancomycin (*n* = 2)	*Enterococcus* species (*n* = 2)	Cow (*n* = 2)	Non-conventional management; Dietary zinc and copper	[38, 50]
*β*-lactams	Ampicillin (*n* = 18)	*E. coli* (*n* = 9), *Salmonella enterica* (*n* = 3), *Campylobacter* species (*n* = 1), coliforms (*n* = 4)	Pig (*n* = 6), cow (*n* = 8); chicken (*n* = 2), dog (*n* = 2)	Non-conventional management; Diet associated with a risk of ruminal acidosis; Bringing new animals onto the farm; Pen/group/herd size; Treatment with oral competitive exclusion culture made from piglets; Education; Oral recombinant *β*-lactamase administration in dogs treated with parenteral ampicillin.	[28, 32–37, 39, 40, 43–45, 47, 50–54]
	Amoxicillin-clavulanic acid (*n* = 7)	*E. coli* (*n* = 4), *Salmonella enterica* (*n* = 3), *Campylobacter* species (*n* = 1)	Pig (*n* = 2), cow (*n* = 3); chicken (*n* = 2)	Non-conventional management; Bringing new animals onto the farm; Pen/group/herd size	[32, 33, 35, 37, 39, 40, 43]
	Carbenicillin (*n* = 1)	Coliforms (*n* = 1)	Pig (*n* = 1)	Non-conventional management	[45]
	Cefoxitin (*n* = 5)	*E. coli* (*n* = 3), *Salmonella enterica* (*n* = 2)	Pig (*n* = 2), cow (*n* = 3), chicken (*n* = 1)	Non-conventional management; Bringing new animals onto the farm; Pen/group/herd size	[32, 33, 39, 43, 50]
	Ceftiofur (*n* = 5)	*E. coli* (*n* = 3), *Salmonella enterica* (*n* = 2)	Pig (*n* = 2), cow (*n* = 2), chicken (*n* = 1)	Non-conventional management; Pen/group/herd size	[33, 35, 39, 43, 54]
	Ceftriaxone (*n* = 7)	*E. coli* (*n* = 3), *Salmonella enterica* (*n* = 3), *Campylobacter* species (*n* = 1), *Enterococcus* species (*n* = 1)	Pig (*n* = 2), cow (*n* = 3), chicken (*n* = 2)	Non-conventional management; Pen/group/herd size	[33, 35, 37, 39, 40, 43, 50]
	Cephalothin (*n* = 8)	*E. coli* (*n* = 4), *Salmonella enterica* (*n* = 3), coliforms (*n* = 1)	Pig (*n* = 2), cow (*n* = 3), chicken (*n* = 2), dog (*n* = 1)	Non-conventional management; Bringing new animals onto the farm; Pen/group/herd size; Oral recombinant *β*-lactamase administration in dogs treated with parenteral ampicillin	[32, 33, 35, 39, 40, 43, 52, 54]
	Penicillin (*n* = 4)	*E. coli* (*n* = 1), *Enterococcus species* (*n* = 2), coliforms (*n* = 1)	Pig (*n* = 1), cow (*n* = 3)	Non-conventional management; Dietary copper and zinc	[38, 44, 50, 54]
	TEM genes (*n* = 1)		Dog (*n* = 1)	Oral recombinant *β*-lactamase administration in dogs treated with parenteral ampicillin	[51]
Carbadox	Carbadox (*n* = 2)	*E. coli* (*n* = 2)	Pig (*n* = 2)	Ration supplemented with egg yolk with anti-*Salmonella* antibody; Bringing new animals onto the farm	[31, 36]
Lipopeptide	Daptomycin (*n* = 1)	*Enterococcus* species (*n* = 1)	Cow (*n* = 1)	Non-conventional management	[50]
Macrolides	Azithromycin (*n* = 2)	*Campylobacter* species (*n* = 2)	Pig (*n* = 1), cow (*n* = 1)	Non-conventional management	[37, 42]
	Erythromycin (*n* = 6)	*E. coli* (*n* = 1), *Campylobacter* species (*n* = 5), *Enterococcus* species (*n* = 1)	Pig (*n* = 3), cow (*n* = 3)	Non-conventional management; *Ad libitum* or restricted feeding; Dietary copper and zinc; Health status	[37, 38, 41, 42, 44, 49, 50]
	Macrolides (*n* = 2)	*E. coli* (*n* = 1)*,Campylobacter* species (*n* = 1)	Pig (*n* = 1), cow (*n* = 1)	Steamed flaked corn with or without wet distillers; Dietary copper	[55, 56]
	Tilmicosin (*n* = 1)	*E. coli* (*n* = 1)	Cow (*n* = 1)	Dietary copper and zinc	[38]
	Tylosin (*n* = 2)	*E. coli* (*n* = 1), *Enterococcus* species (*n* = 1)	Cow (*n* = 2)	Non-conventional management; Dietary copper and zinc	[38, 50]
	ermB gene (*n* = 1)	Coliforms (*n* = 1)	Cow (*n* = 1)	Steamed flaked corn with or without wet distillers	[55]
Nitrofurans	Nitrofurantoin (*n* = 1)	*Enterococcus* species (*n* = 1)	Pig (*n* = 1)	Pen/group/herd size; Bringing new animals onto a farm	[36]
Oxazolidinones	Linezolid (*n* = 2)	*Enterococcus* species (*n* = 2)	Cow (*n* = 2)	Non-conventional management; Dietary copper and zinc	[38, 50]
Phenicols	Chloramphenicol (*n* = 10)	*E. coli* (*n* = 5), *Enterococcus* species (*n* = 2), *Salmonella enteria* (*n* = 2), *Campylobacter* species (*n* = 2)	Cow (*n* = 6), pig (*n* = 3), chicken (*n* = 1)	Non-conventional management; Dietary copper and zinc; Pen/group/herd size; Number of suppliers; Bringing new animals onto the farm	[32, 35, 37–41, 43, 47, 50]
	Florfenicol (*n* = 1)	*Campylobacter* species (*n* = 1)	Cow (*n* = 1)	Non-conventional management	[37]
Phosphoglycolipid	Flavomycin (*n* = 2)	*Campylobacter* species (*n* = 1), *Enterococcus* species (*n* = 1)	Cow (*n* = 2)	Non-conventional management; Steamed corn with or without wet distillers	[50, 55]
Pleuromutilin	Tiamulin (*n* = 1)	*E. coli* (*n* = 2)	Cow (*n* = 2)	Dietary copper and zinc	[38]
Quinlones/Fluoroquinolones	Ciprofloxacin (*n* = 8)	*E. coli* (*n* = 3), *Campylobacter* species (*n* = 4), *Enterococcus* species (*n* = 1)	Pig (*n* = 5), cow (*n* = 3)	Non-conventional management; All-in/all-out system; Dietary copper and zinc; Ration with or without whey; *Ad libitum* or restricted feeding Health status; Disinfection; Rodent control	[35, 37–39, 41–43, 49]
	Nalidixic acid (*n* = 7)	*E. coli* (*n* = 3), *Campylobacter* species (*n* = 4)	Pig (*n* = 4), cow (*n* = 3)	Non-conventional management; Pen/group/herd size	[35, 37, 39, 41–43, 47]
Streptogamins	Quinpristin/dalfopristin (*n* = 1)	*Campylobacter* species (*n* = 1)	Cow (*n* = 1)	Steamed flaked corn with or without wet distillers	[55]
Sulphonamides/Pyrimidines	Sulfadimethoxine (*n* = 2)	*E. coli* (*n* = 2)	Cow (*n* = 2)	Non-conventional management; Steamed flaked corn with or without wet distillers	[54, 55]
	Sulfamethizole (*n* = 1)	Coliforms (*n* = 1)	Pig (*n* = 1)	Non-conventional management	[44]
	Sulfamethoxazole (*n* = 6)	*E. coli* (*n* = 5), *Campylobacter* species (*n* = 1)	Pig (*n* = 2), cow (*n* = 4)	Non-conventional management; Pen/group/herd size; Number of supplies; Bringing new animals onto the farm; Education	[32, 35, 37, 39, 43, 47]
	Sulfasoxazole (*n* = 3)	*E. coli (n* = 2), *Salmonella enterica* (*n* = 1), coliforms (*n* = 1)	Pig (*n* = 2), chicken (*n* = 1)	Non-conventional management; Pen/group/herd size; Bringing new animals onto the farm	[40]
	Sulfathiazole (*n* = 1)	*E. coli* (*n* = 1)	Cow (*n* = 1)	Steamed flaked corn with or without wet distillers	[55]
	Sulfachloropyridazine (*n* = 1)	*E. coli* (*n* = 1)	Cow (*n* = 1)	Steamed flaked corn with or without wet distillers	[55]
	Trimethoprim- sulfamethoxazole (*n* = 6)	*E. coli* (*n* = 5), *Salmonella enterica* (*n* = 1), coliforms (*n* = 1)	Pig (*n* = 2), cow (*n* = 2), dog (*n* = 1)	Non-conventional management; Pen/group/herd size; Number of suppliers; Education; Bringing new animals onto the farm; Oral recombinant *β*-lactamase administration in dogs treated with parenteral ampicillin	[32, 35, 39, 43, 47, 52]
Tetracyclines	Tetracycline (*n* = 21)	*E. coli* (*n* = 11), *Salmonella enterica* (*n* = 3), *Campylobacter* species (*n* = 6), *Enterococcus* species (*n* = 1), coliforms (*n* = 1)	Pig (*n* = 12), cow (*n* = 6), chicken (*n* = 2), dog (*n* = 1)	Non-conventional management; All-in/all-out; Ventilation system; Contact with animals treated with antimicrobials; Disinfection and sanitation; Ration supplemented with egg yolk with anti-*Salmonella* antibody; Flooring; Pen/group/herd size; Diet associated with a risk of ruminal acidosis; Education; Bringing new animals onto the farm; Number of suppliers; Treatment with oral competitive exclusion culture made from piglets; Oral recombinant *β*-lactamase administration in dogs treated with parenteral ampicillin; Health status; Silage *vs.* a grain based ration.	[28, 30–37, 39–45, 47, 49, 50, 52–54, 57, 58]
	tetM gene (*n* = 1)		Cow (*n* = 1)	Steamed flaked corn with or without wet distillers	[55]
Other	Any resistance (*n* = 5)	*E. coli* (*n* = 3), *Salmonella enterica* (*n* = 1), coliforms (*n* = 1)	Cow (*n* = 3), pig (*n* = 2)	Non-conventional management; All-in/all-out; Pen/group/herd size; Health status	[45, 59–62]
	Multiple resistance‡ (*n* = 15)	*E. coli, Salmonella enterica, Campylobacter* species, coliforms	Cow (*n* = 6), pig (*n* = 6), chicken (*n* = 2), dog (*n* = 1)	Non-conventional management; Health status; All-in/all-out; *Ad libitum* or restricted feeding; Disinfection and sanitation; Bringing new animals onto the farm; Mixing of animals from different sources; Pen/group/herd size; Oral recombinant *β*-lactamase administration in dogs treated with parenteral ampicillin; Addition of live *S. cerevisisae boulardii* to milk replacer	[32, 33, 35, 40–42, 44, 45, 49, 52, 61, 63–66]

* Number of citations.

† The following management systems as defined in the studies were aggregated under non-conventional management systems: organic, antimicrobial-free, extensive (e.g. pasture raised pigs), natural production, animal friendly or ecological.

‡ Multiple drug resistance was captured from studies where multiple drug resistance was defined and investigated, as well as any study where susceptibility two or more antimicrobials in combination were reported (excluding trimethoprim-sulfamethoxazole and quinpristin-dalfopristin).

**Table 2.** The most frequently* identified bacteria/antimicrobial/species combinations reported from retained citations (n = 39) in animal populations that investigated the associations between modifiable non-antimicrobial factors or interventions and antimicrobial resistance.

Combination	Animal	No.	Frequency*
*E. coli*/tetracycline		26	5%
	Pig	17	
	Cow	9	
*E*. *coli*/ampicillin		17	4%
	Pig	10	
	Cow	7	
*E*. *coli*/apramycin		15	3%
	Pig	12	
	Cow	3	
*Campylobacter*/ciprofloxacin		15	3%
	Pig	14	
	Cow	1	
*E*. *coli*/gentamicin		13	3%
	Pig	8	
	Cow	5	
*E*. *coli*/streptomycin		13	3%
	Pig	6	
	Cow	7	

* Denominator was total number of associations reported in ‘reliable’ studies (*n* = 485). All other bacteria/antimicrobial combinations were identified at a frequency of < 2%.

Specific associations between a given factor or intervention with antimicrobial resistance in a given animal population (e.g. the association between non-conventional management and tetracycline susceptibility in *E. coli* from cattle) were reported in only a small number (range 1–3) of studies. The majority (*n* = 303, 62%) of specific associations were reported in only one study, 24% (*n* = 115) were reported in two studies, and 14% (*n* = 67) were reported in three studies.

Most reported associations between antimicrobial susceptibility and a modifiable non-antimicrobial factor or intervention were not statistically significant (60%, *n* = 290), 27% (*n* = 132) (27%) of the associations were interpreted as significant [*P*⩽0·05 and confidence interval (where reported) that excluded 1] and the remainder of the associations that were reported (*n* = 63, 13%) were descriptive in nature or else raw data (Table 3). Use of a non-conventional management systems (e.g. organic, antimicrobial-free, extensive systems), increasing herd, group or pen size and all-in/all-out systems were the factors with the greatest number of reported associations (*n* = 183, 68 and 52, respectively). For the factors of non-conventional management systems and increasing herd, group or pen, most associations were non-significant (64% and 90%, respectively); however, the majority (83%) of the associations for all-in/all-out systems were significantly associated with a decrease in antimicrobial resistance.

**Table 3.** Direction of effect of associations between modifiable non-antimicrobial factors or interventions and antimicrobial resistance in retained citations (n = 39) in animal populations

Factor or intervention	Species	Increase antimicrobial resistance (no. of associations)	Decrease antimicrobial resistance (no. of associations)	Not statistically associated with antimicrobial resistance (no. of associations)
Contact with animals				
Contact with animals treated with antimicrobials	Pig	1		3
Diet				
Addition of egg yolk anti- *Salmonella* antibody to a ration	Pig		8	7
Addition of whey to a ration	Pig		1	
*Ad libitum* feeding when compared to restricted feeding	Pig	1		
Addition of wet distillers to a ration of steamed flaked corn	Cow	3	1	4
A diet associated with a low risk of ruminal acidosis	Cow		1	1
Addition of *S*. *cerevisisae boulardii* to milk replacer	Cow	1		1
Dietary zinc or copper above NRC recommendation	Cow	1		26
Health status				
Unhealthy animals	Cow, pig	5	1	
Routine deworming	Pig		1	
Hygiene				
Dirty pens	Pig	2		1
Management				
Non-conventional management systems (e.g. organic, antimicrobial-free, extensive)	Chicken, cow, pig	2	63	118
All-in/all-out systems	Pig	3	43	6
Increasing pen, group or herd size	Cow, pig	7		61
Fully slatted floors compared to partially slatted	Pig	1		1
Heat or cold stress	Pig	2		
Education				
Participation in a quality assurance programme	Cow		5	
Non-antimicrobial therapies				
Treatment with an oral competitive exclusion derived from the intestinal tracts of neonatal pigs	Pig	3		5
Oral recombinant *β*-lactamase with parenteral administration of ampicillin	Dog		3	6

NRC, National Research Council Canada.

In the retained studies from human and *in vitro* populations (*n* = 424) (Table 4), most factors were associated with an increase, rather than decrease in antimicrobial resistance. The most frequently reported factor (*n* = 289, 68%) was the duration of hospitalization (or time spent in the intensive care unit or in another ward).

**Table 4.** Distribution of categories of modifiable non-antimicrobial factors or inventions described retained citations in human populations (n = 356) where there was a statistically significant association with antimicrobial resistance

Category	Factors or intervention	Decrease	Increase
Biocides or disinifectants	Antiseptic soap or hand sanitizers, benzalkonium, biocides, chlorhexidine, disinfectant, ethdium bromide, house cleaning solution, pine oil, triclosan	0	5
Hospital-related factors	Increasing length of hospitalization or time spent in an intensive care unit or other ward	0	289
	Colonization pressure	0	8
	Daycare	0	3
	Infectious disease consultation	1	1
	Interval between hospitalizations	0	0
	Long-term care	1	8
	Hospital size	0	1
	Multiple visits to a hospital	0	0
	Overcrowding	0	2
	Transfers between units	0	11
Individual-level factors	Aspirin/salicylic acid	0	3
	Breast milk	1	0
	Hamycin treatment	0	0
	Immunosuppresives	1	13
	*Lactobacillus* inocula	0	0
	Laparotomy *vs*. laproscopy	0	0
	Pneumonoccal vaccination	14	0
	Sucralfate	0	2
			
Total citations		18	364

Considering combined data from human, animal and *in vitro* populations, 16 common themes were observed (Table 5). These themes reflected aspects on the use of compounds (e.g. vaccinations, probiotics, and recombinant *b*-lactamases), infection control (e.g. cleaning and disinfection, hospital *vs.* home pens), health (comorbidity, stressors, immunosuppression), education and relationships with professionals (infection disease consultations, participation in quality assurance programmes) and others. Studies in animal populations were represented in all themes despite fewer studies retained (*n* = 39) compared to retained studies in human populations (*n* = 356) or entirely *in vitro* studies (*n* = 68). Studies performed in human populations or entirely *in vitro* belonged to only 50% (*n* = 8) of the themes and belonged to themes identified in animal populations.

**Table 5.** Common themes of modifiable non-antimicrobial factors or interventions reported in citations from retained animal, human or in vitro populations

	Factors from	
Common themes	Human or *in vitro* populations (*n* = 356 references)	Animal populations (*n* = 39 references)
Cleaning and disinfection	Various biocides, disinfectants and hand cleaners/sanitizers	Cleaning and disinfection, hygiene and sanitation
Relationships with professionals	Infectious disease consultation, multiple visits to a hospital	Participation in a quality assurance programme
Hospital *vs*. home pens	Colonization pressure, daycare, interval between hospitalizations, residence in LTC, hospital size, crowding, transfer between units	Herd/pen/group size, crowding, contact with animals treated with antimicrobials
Probiotic	*Lactobacillus* inocula	*S*. *cerevisiae boulardii*, porcine competitive exclusion culture
Vaccination	Pneumococcal vaccination	Anti-*Salmonella* antibody
Housing		Ventilation and flooring systems
Non-conventional management systems		ABF, organic, animal friendly, ecological
Temperature stress		Cold/heat stress
Minerals		Zinc and copper
Recombinant *β*-lactamases		Recombinant *β*-lactamase
Health status	Immunosuppressives	Health status, comorbidity, routine deworming
Gastric/ruminal pH	Sucralfate	High *vs*. low pH
Availability of feed		*Ad libitum vs*. restricted
Energy sources		Corn (dry, rolled, flaked steamed), wet distillers, whey
Feed form		Pelleted, mash, liquid
Group size	Hospital size	Group/pen/herd size

LTC, Long-term care; ABF, antibiotic free.

## DISCUSSION

Using scoping review methodology, we identified the evidence supporting use of non-antimicrobial factors or interventions for reduction of antimicrobial resistance with particular emphasis on animal populations. We also assessed the quantity and quality of this evidence, as well as the distribution of antimicrobials selected for susceptibility testing in enteric bacteria, the distribution of enteric bacteria investigated and the populations studied (animal, human, *in vitro*) in a transparent, reproducible manner.

An important finding of this review was the wide breadth of data on non-antimicrobial factors associated with antimicrobial resistance; however, the depth of research supporting effect measures for these factors was limited, particularly concerning any specific factor for any particular antimicrobial/bacterial combination in any particular animal population. The limited depth of applicable research in animal populations is perhaps somewhat offset by similar research in human populations, from which some analogies may be drawn.

Another key finding of this review was that most of the non-antimicrobial factors or interventions tested in animal populations were not significantly associated with antimicrobial resistance. Additionally, where significant associations were reported, they were not generally in a single direction (either associated with an increase or decrease in the occurrence of antimicrobial resistance, or both). In light of the general paucity of research in this area, we did not identify any modifiable non-antimicrobial factors or interventions that could on the basis of available evidence be strongly recommended for consideration, application or adoption by the North American cattle industry without additional research, or unless the factor(s) or intervention(s) have other well established positive industry impacts and would likely not propagate antimicrobial resistance.

When considering together the results of studies in animal and human populations, some non-antimicrobial factors (e.g. health management, improving sanitation) may be sufficiently well-established principles for disease prevention that they may not need additional research; that is, before veterinarians and beef producers could be convinced of the value in adoption by the North American cattle industry. Some of these factors (e.g. diet, reducing pen size, heat/cold stressors, limiting contact with animals treated with antimicrobials, reducing time in a hospital pen) may also result in other positive outcomes (i.e. improved animal health and welfare, public health, food safety) in addition to reducing antimicrobial resistance. To the extent that implementation of such disease prevention strategies reduces the need for antimicrobial treatment, benefits in reduced resistance selection pressure could indirectly arise. However, adoption by sectors of the cattle industry may be technically or practically more difficult and require additional resources, changes to facility design, management style, or systems. Regardless, additional research is needed to adequately demonstrate the efficacy of any of these factors to reduce the occurrence of antimicrobial resistance, either as a whole or in specific ‘bug/drug’ combinations, in public health or animal health settings.

One specific intervention that has been studied in human populations, but not to our knowledge in cattle populations, is the use of vaccination to address resistance concerns in bacterial populations. There is good evidence that pneumococcal vaccination reduces antimicrobial resistance in pneumococcal bacteria in humans. This finding has been attributed to a reduced incidence of disease and a shift to less phenotypically resistant pneumococcal strains. In cattle studies, vaccination for bovine respiratory disease complex or undifferentiated fever was associated with reduced frequency of respiratory disease and systemic antimicrobial treatment [22, 23]; however, effects on antimicrobial susceptibility were not reported. It would be helpful if future studies of vaccine efficacy in food animals included evaluation of effect on antimicrobial resistance in both the pathogen of interest and commensal bacteria in target (e.g. lung) and non-target sites, such as the gastrointestinal tract.

Several knowledge gaps were identified concerning non-antimicrobial interventions against resistance. For example, no studies were found on the impact on resistance of some common North American cattle industry practices such as preconditioning (as a whole) or factors associated with preconditioning (e.g. vaccination, reducing stressors associated with weaning, transportation, feed introduction), the impact of different bedding packs and materials, the use of hospital or sick pens *vs.* ‘treat and go-home’ practices, impact of veterinary consultations, and other educational actions directed at producers. Factors such as mixing groups of treated and untreated animals, open *vs.* closed population management, and all-in/all-out systems that have been studied in other animal species (e.g. pigs) need to be assessed for relevance to the various cattle production sectors. Associations between biosecurity measures in cattle production and antimicrobial resistance are also areas worthy of additional research, based on the findings in human populations related to time spent in the hospital or intensive care wards.

Although the findings of this review may be limited by the decision to not update from 2011 the searches during the review process, it has been reported that updates to *Cochrane Reviews* altered conclusions in only 9% of updated reviews [24, 25]. Our review included comprehensive searches (multiple databases and all years searched) and, a large number of citations screened (27 060). To our knowledge, similar reviews have not been published. The findings provide a baseline for evidence-based recommendations on interventions to reduce the occurrence of antimicrobial resistance, identify areas for future research including knowledge gaps and establish a framework upon which data from more recent and future studies can be added.

The findings may also have been limited by the decision to include only English language publications, but the impact of this is difficult to determine [26, 27]; other agri-food reviews have reported that the number of relevant non-English-language citations were few and therefore unlikely to impact findings [25]. As many of the countries with influential research on antimicrobial resistance publish in English-language peer-reviewed journals or provide other documents in English, this potential bias was likely small.

## CONCLUSION

A diverse but shallow body of evidence presents investigated factors or interventions associated with antimicrobial resistance and the addition of human studies strengthened the data reported in animal populations. However, the results of this review could not identify any modifiable non-antimicrobial factors or interventions that could be recommended for consideration, application or adoption by the North American cattle industry without additional research. Research is needed to more comprehensively study these interventions on the occurrence of antimicrobial resistance as a whole and in specific ‘bug/drug’ combinations of public and animal health interest.

## Supplementary material

For supplementary material accompanying this paper visit http://doi.org/10.1017/S0950268815000722. click here to view supplementary material

## References

[1] Forbes GB. Infection with penicillin-resistant staphylococci in hospital and general practice. British Medical Journal 1949; 2: 569–571. 18139450 10.1136/bmj.2.4627.569 PMC2051151

[2] Seiffert SN, Extended-spectrum cephalosporin-resistant Gram-negative organisms in livestock: an emerging problem for human health? Drug Resistance Updates 2013; 16: 22–45. 23395305 10.1016/j.drup.2012.12.001

[3] Guerra B, Fischer J, Helmuth R. An emerging public health problem: acquired carbapenemase-producing microorganisms are present in food-producing animals, their environment, companion animals and wild birds. Veterinary Microbiology 2014; 171: 290–297. 24629777 10.1016/j.vetmic.2014.02.001

[4] Woodford N, Carbapenemase-producing enterobacteriaceae and non-enterobacteriaceae from animals and the environment: an emerging public health risk of our own making? Journal of Antimicrobial Chemotherapy 2014; 69: 287–291. 24092657 10.1093/jac/dkt392

[5] Varma JK, Highly resistant *Salmonella* Newport-MDRAmpC transmitted through the domestic US food supply: a FoodNet case-control study of sporadic Salmonella Newport infections, 2002–2003. Journal of Infectious Diseases 2006; 194: 222–230. 16779729 10.1086/505084

[6] Hummel R, Tschape H, Witte W. Spread of plasmid-mediated nourseothricin resistance due to antibiotic use in animal husbandry. Journal of Basic Microbiology 1986; 26: 461–466. 3033194 10.1002/jobm.3620260806

[7] Witte W. Impact of antibiotic use in animal feeding on resistance of bacterial pathogens in humans. Ciba Foundation Symposium 1997; 207: 61–71. 9189635 10.1002/9780470515358.ch5

[8] Davies R, Roberts TA. Antimicrobial susceptibility of enterococci recovered from commercial swine carcasses: effect of feed additives. Letters in Applied Microbiology 1999; 29: 327–333. 10664974 10.1046/j.1472-765x.1999.00634.x

[9] Food and Drug Administration, Center for Veterinary Medicine. The human health impact of fluoroquinolone resistant *Campylobacter* attributed to the consumption of chicken. 2001. (http://www.fda.gov/downloads/AnimalVeterinary/SafetyHealth/RecallsWithdrawals/UCM042038.pdf) Accessed 10 September 2014.

[10] Threlfall EJ. Antimicrobial drug resistance in *Salmonella*: problems and perspectives in food- and water-borne infections. FEMS Microbiology Reviews 2002; 26: 141–148. 12069879 10.1111/j.1574-6976.2002.tb00606.x

[11] Cheng AC, Control of fluoroquinolone resistance through successful regulation, Australia. Emerging Infectious Diseases 2012; 18: 1453–1460. 22932272 10.3201/eid1809.111515 PMC3437704

[12] Gupta A, Antimicrobial resistance among *Campylobacter* strains, United States, 1997–2001. Emerging Infectious Diseases 2004; 10: 1102–1109. 15207064 10.3201/eid1006.030635 PMC3323172

[13] Dutil L, Ceftiofur resistance in *Salmonella enterica* serovar Heidelberg from chicken meat and humans, Canada. Emerging Infectious Diseases 2010; 16: 48–54. 20031042 10.3201/eid1601.090729 PMC2874360

[14] Otto SJ, Estimating the number of human cases of ceftiofur-resistant *Salmonella enterica* serovar Heidelberg in Quebec and Ontario, Canada. *Clinical Infectious Diseases* 2014; 59: 1281–1290. 10.1093/cid/ciu496 24982036

[15] Danish Antimicrobial Integrated Antimicrobial Resistance Monitoring and Research Programme (DANMAP). Data for action. The Danish approach to surveillance of the use of antimicrobial agents and the occurrence of antimicrobial resistance in bacteria from food animals, food and humans in Denmark. 2014. (http://www.danmap.org/~/media/Projekt%20sites/Danmap/DivDownloads/Data_for_action.ashx). Accessed 10 September 2014.

[16] Pham MT, The utility of systematic reviews for informing agri-food public health policy: a survey of Canadian policy makers. In: *21st Cochrane Colloquium, 19–23 September 2013*, Quebec City, Canada, 2013, pp. P2·074.

[17] Wilhelm BJ, A systematic review/meta-analysis of primary research investigating swine, pork or pork products as a source of zoonotic hepatitis E virus. Epidemiology and Infection 2011; 139: 1127–1144. 21554782 10.1017/S0950268811000677

[18] Bucher O, Evaluating interventions against *Salmonella* in broiler chickens: applying synthesis research in support of quantitative exposure assessment. Epidemiology and Infection 2012; 140: 925–945. 21781371 10.1017/S0950268811001373

[19] Tusevljak N, Prevalence of zoonotic bacteria in wild and farmed aquatic species and seafood: a scoping study, systematic review, and meta-analysis of published research. Foodborne Pathogens and Disease 2012; 9: 487–497. 22571642 10.1089/fpd.2011.1063

[20] Arksey H, O'Malley L. Scoping studies: towards a methodological framework. International Journal of Social Research Methodology 2005; 8: 19–32.

[21] Guyatt GH, GRADE guidelines: 4. Rating the quality of evidence – study limitations (risk of bias). Journal of Clinical Epidemiology 2011; 64: 407–415. 21247734 10.1016/j.jclinepi.2010.07.017

[22] Makoschey B, Field efficacy of combination vaccines against bovine respiratory pathogens in calves. Acta Veterinaria Hungarica 2008; 56: 485–493. 19149103 10.1556/AVet.56.2008.4.6

[23] Booker CW, Seroepidemiology of undifferentiated fever in feedlot calves in western Canada. Canadian Veterinary Journal 1999; 40: 40–48. PMC1539654 9919366

[24] French SD, Investing in updating: how do conclusions change when Cochrane systematic reviews are updated? BMC Medical Research Methodology 2005; 5: 33. 16225692 10.1186/1471-2288-5-33 PMC1274326

[25] Young I, The application of knowledge synthesis methods in agri-food public health: recent advancements, challenges and opportunities. Preventive Veterinary Medicine 2014; 113: 339–355. 24485274 10.1016/j.prevetmed.2013.11.009

[26] Moher D, The inclusion of reports of randomised trials published in languages other than English in systematic reviews. Health Technology Assessment 2003; 7: 1–90. 10.3310/hta7410 14670218

[27] Juni P, Direction and impact of language bias in meta-analyses of controlled trials: empirical study. International Journal of Epidemiology 2002; 31: 115–123. 11914306 10.1093/ije/31.1.115

[28] Kim LM, Effect of porcine-derived mucosal competitive exclusion culture on antimicrobial resistance in *Escherichia coli* from growing piglets. Foodborne Pathogens & Disease 2005; 2: 317–329. 16366854 10.1089/fpd.2005.2.317

[29] Mathew AG, Characterization of resistance patterns and detection of apramycin resistance genes in *Escherichia coli* isolated from swine exposed to various environmental conditions. International Journal of Food Microbiology 2003; 89: 11–20. 14580969 10.1016/s0168-1605(03)00124-7

[30] Mathew AG, Effects of antibiotic use in sows on resistance of *E*. *coli* and *Salmonella enterica* Typhimurium in their offspring. Foodborne Pathogens & Disease 2005; 2: 212–220. 16156702 10.1089/fpd.2005.2.212

[31] Mathew AG, Effects of in-feed egg yolk antibodies on *Salmonella* shedding, bacterial antibiotic resistance, and health of pigs. Journal of Food Protection 2009; 72: 267–273. 19350971 10.4315/0362-028x-72.2.267

[32] Sato K, Bartlett PC, Saeed MA. Antimicrobial susceptibility of *Escherichia coli* isolates from dairy farms using organic versus conventional production methods. Journal of the American Veterinary Medical Association 2005; 226: 589–594. 15742702 10.2460/javma.2005.226.589

[33] Alali WQ, Prevalence and distribution of *Salmonella* in organic and conventional broiler poultry farms. Foodborne Pathogens & Disease 2010; 7: 1363–1371. 20617937 10.1089/fpd.2010.0566

[34] Alexander TW, Effect of subtherapeutic administration of antibiotics on the prevalence of antibiotic-resistant *Escherichia coli* bacteria in feedlot cattle. Applied & Environmental Microbiology 2008; 74: 4405–4416. 18502931 10.1128/AEM.00489-08 PMC2493153

[35] Bunner CA, Prevalence and pattern of antimicrobial susceptibility in *Escherichia coli* isolated from pigs reared under antimicrobial-free and conventional production methods. Journal of the American Veterinary Medical Association 2007; 231: 275–283. 17630898 10.2460/javma.231.2.275

[36] Dunlop RH, Associations among antimicrobial drug treatments and antimicrobial resistance of fecal *Escherichia coli* of swine on 34 farrow-to-finish farms in Ontario, Canada. Preventive Veterinary Medicine 1998; 34: 283–305. 9618742 10.1016/s0167-5877(97)00095-0

[37] Halbert LW, Evaluation of antimicrobial susceptibility patterns in *Campylobacter* spp isolated from dairy cattle and farms managed organically and conventionally in the midwestern and northeastern United States. Journal of the American Veterinary Medical Association 2006; 228: 1074–1081. 16579787 10.2460/javma.228.7.1074

[38] Jacob ME, Effects of feeding elevated concentrations of copper and zinc on the antimicrobial susceptibilities of fecal bacteria in feedlot cattle. Foodborne Pathogens & Disease 2010; 7: 643–648. 20482227 10.1089/fpd.2009.0401

[39] Morley PS, Effects of restricted antimicrobial exposure on antimicrobial resistance in fecal *Escherichia coli* from feedlot cattle. Foodborne Pathogens & Disease 2011; 8: 87–98. 21034271 10.1089/fpd.2010.0632

[40] Siemon CE, Bahnson PB, Gebreyes WA. Comparative investigation of prevalence and antimicrobial resistance of *Salmonella* between pasture and conventionally reared poultry. Avian Diseases 2007; 51: 112–117. 17461275 10.1637/0005-2086(2007)051[0112:CIOPAA]2.0.CO;2

[41] Tadesse DA, Prevalence and antimicrobial resistance profile of *Campylobacter* spp. isolated from conventional and antimicrobial-free swine production systems from different U.S. regionsp. Foodborne Pathogens and Disease 2011; 8: 367–374. 21133777 10.1089/fpd.2010.0665

[42] Rollo SN, Prevalence and patterns of antimicrobial resistance in *Campylobacter* spp isolated from pigs reared under antimicrobial-free and conventional production methods in eight states in the Midwestern United States. Journal of the American Veterinary Medical Association 2010; 236: 201–210. 20074013 10.2460/javma.236.2.201

[43] Rosengren LB, Antimicrobial resistance of fecal *Escherichia coli* isolated from grow-finish pigs in 20 herds in Alberta and Saskatchewan. Canadian Journal of Veterinary Research 2008; 72: 160–167. 18505205 PMC2276901

[44] Dawson KA, Multiple antibiotic resistance in fecal, cecal and colonic coliforms from pigs fed therapeutic and subtherapeutic concentrations of chlortetracycline. Journal of Animal Science 1983; 57: 1225–1234. 6643317 10.2527/jas1983.5751225x

[45] Langlois BE, Effect of age and housing location on antibiotic resistance of fecal coliforms from pigs in a non-antibiotic-exposed herd. Applied & Environmental Microbiology 1988; 54: 1341–1344. 2970822 10.1128/aem.54.6.1341-1344.1988 PMC202660

[46] Akwar HT, Associations of antimicrobial uses with antimicrobial resistance of fecal *Escherichia coli* from pigs on 47 farrow-to-finish farms in Ontario and British Columbia. Canadian Journal of Veterinary Research 2008; 72: 202–210. 18505211 PMC2276907

[47] Di Labio E, Antimicrobial resistance in bacteria from Swiss veal calves at slaughter. Zoonoses & Public Health 2007; 54: 344–352. 18035972 10.1111/j.1863-2378.2007.01071.x

[48] Kim JY, Control of extended-spectrum *β*-lactamase-producing *Klebsiella pneumoniae* using a computer-assisted management program to restrict third-generation cephalosporin use. Journal of Antimicrobial Chemotherapy 2008; 62: 416–421. 18413317 10.1093/jac/dkn164

[49] Schuppers ME, Clinical herd health, farm management and antimicrobial resistance in *Campylobacter coli* on finishing pig farms in Switzerland. Preventive Veterinary Medicine 2005; 69: 189–202. 15907569 10.1016/j.prevetmed.2005.02.004

[50] Zhang J, Contamination rates and antimicrobial resistance in bacteria isolated from ‘grass-fed’ labeled beef products. Foodborne Pathogens & Disease 2010; 7: 1331–1336. 20618073 10.1089/fpd.2010.0562

[51] Harmoinen J, Orally administered targeted recombinant beta-lactamase prevents ampicillin-induced selective pressure on the gut microbiota: a novel approach to reducing antimicrobial resistance. Antimicrobial Agents & Chemotherapy 2004; 48: 75–79. 14693521 10.1128/AAC.48.1.75-79.2004 PMC310163

[52] Mentula S, Inhibition of ampicillin-induced emergence of resistance in intestinal coliforms by targeted recombinant beta-lactamase. International Journal of Antimicrobial Agents 2004; 24: 555–561. 15555877 10.1016/j.ijantimicag.2004.07.008

[53] Penner G, Commensal fecal *Escherichia coli* diversity in dairy cows at high and low risk for incurring subacute ruminal acidosis. Foodborne Pathogens and Disease 2009; 6: 973–980. 19642917 10.1089/fpd.2009.0270

[54] Pol M, Ruegg PL. Relationship between antimicrobial drug usage and antimicrobial susceptibility of gram-positive mastitis pathogens. Journal of Dairy Science 2007; 90: 262–273. 17183094 10.3168/jds.S0022-0302(07)72627-9

[55] Jacob ME, Effects of feeding wet corn distillers grains with solubles with or without monensin and tylosin on the prevalence and antimicrobial susceptibilities of fecal foodborne pathogenic and commensal bacteria in feedlot cattle. Journal of Animal Science 2008; 86: 1182–1190. 18192558 10.2527/jas.2007-0091

[56] Hasman H, Copper resistance in *Enterococcus faecium*, mediated by the tcrB gene, is selected by supplementation of pig feed with copper sulfate. Applied & Environmental Microbiology 2006; 72: 5784–5789. 16957194 10.1128/AEM.02979-05 PMC1563648

[57] Dewulf J, Tetracycline-resistance in lactose-positive enteric coliforms originating from Belgian fattening pigs: degree of resistance, multiple resistance and risk factors. Preventive Veterinary Medicine 2007; 78: 339–351. 17156871 10.1016/j.prevetmed.2006.11.001

[58] Sato K, Comparison of prevalence and antimicrobial susceptibilities of *Campylobacter* spp. isolates from organic and conventional dairy herds in Wisconsin. Applied & Environmental Microbiology 2004; 70: 1442–1447. 15006764 10.1128/AEM.70.3.1442-1447.2004 PMC368295

[59] Farzan A, Evaluation of the risk factors for shedding *Salmonella* with or without antimicrobial resistance in swine using multinomial regression method. Zoonoses & Public Health 2010; 57: 85–93. 21083821 10.1111/j.1863-2378.2010.01357.x

[60] Gow SP, Prevalence of antimicrobial resistance in fecal generic *Escherichia coli* isolated in western Canadian cow-calf herds. Part I – beef calves. Canadian Journal of Veterinary Research 2008; 72: 82–90. 18505196 PMC2276909

[61] Kaneene JB, Changes in multidrug resistance of enteric bacteria following an intervention to reduce antimicrobial resistance in dairy calves. Journal of Clinical Microbiology 2009; 47: 4109–4112. 19846639 10.1128/JCM.01939-09 PMC2786622

[62] Walk ST, Influence of antibiotic selection on genetic composition of *Escherichia coli* populations from conventional and organic dairy farms. Applied & Environmental Microbiology 2007; 73: 5982–5989. 17704272 10.1128/AEM.00709-07 PMC2074991

[63] Adhikari B, The role of animal movement, including off-farm rearing of heifers, in the interherd transmission of multidrug-resistant *Salmonella*. Journal of Dairy Science 2009; 92: 4229–4238. 19700684 10.3168/jds.2008-1494

[64] Berge AC, Geographic, farm, and animal factors associated with multiple antimicrobial resistance in fecal *Escherichia coli* isolates from cattle in the western United States. Journal of the American Veterinary Medical Association 2010; 236: 1338–1344. 20550450 10.2460/javma.236.12.1338

[65] Berge AC, Moore DA, Sischo WM. Prevalence and antimicrobial resistance patterns of *Salmonella enterica* in preweaned calves from dairies and calf ranches. American Journal of Veterinary Research 2006; 67: 1580–1588. 16948605 10.2460/ajvr.67.9.1580

[66] Galvao KN, Effect of feeding live yeast products to calves with failure of passive transfer on performance and patterns of antibiotic resistance in fecal *Escherichia coli*. Reproduction, Nutrition, Development 2005; 45: 427–440. 16045891 10.1051/rnd:2005040

