# Supplementary material for: Scoping review to identify potential non-antimicrobial interventions to mitigate antimicrobial resistance in commensal enteric bacteria in North American cattle production systems
Source: Epidemiol Infect. 2015 Apr 23;144(1):1–18. doi: 10.1017/S0950268815000722 (PMC4697299; doi:10.1017/S0950268815000722)
Supplement: Supplementary file 1 [file S0950268815000722sup001.doc]

**SUPPLEMENTARY MATERIAL**

Appendix 1. Search terms to return references in animal populations for screening in a scoping review to identify modifiable factors or interventions to reduce the occurrence of antimicrobial resistance in cattle production systems.

1. exp Drug Resistance, Microbial/ or exp Drug Resistance, Bacterial/ or exp Drug Resistance, Multiple, Bacterial/

2. ((antibiotic$ or antimicrobial or aminoglycoside$ or Amphenicol$ or cephalosporin$ or glycopeptide$ or lincosamide$ or spectinomycin$ or macrolide$ or nitrofuran$ or novobiocin$ or oxazolodinone$ or Penicillin$ or Quinolone$ or rifamycin$ or streptogamin$ or sulfonamide$ or trimethoprim$ or Tetracycline$) adj2 (resistanc$ or sensitivit$ or susceptib$)).ti,ab.

3. 1 or 2

4. exp Feces/

5. (feces or fecal or enteric$ or commensal$).ti,ab.

6. 4 or 5

7. exp cattle/

8. (cow$ or cattle or bovine$ or calf or calves or heifer$ or steer$ or beef$).ti,ab.

9. exp Swine/

10. (pig or pigs or sow or sows or weaner$ or hog or hogs or barrow or barrows or boar or boars or porker$ or swine or porcine).ti,ab.

11. exp Poultry/

12. (chick$ or hatchling$ or poultry or turkey$ or pullet$ or broiler$ or fryer$ or capon$ or hen or hens or rooster$ or fowl or fowls).ti,ab.

13. exp deer/ or exp goats/ or exp sheep/

14. (sheep or ewe or ewes or ram or rams or lamb or lambs or goat$ or ramling or wether$ or ovine or caprine or (small adj1 ruminant$)).ti,ab.

15. exp Horses/

16. (horse or horses or mare or mares or foal$ or stallion$ or gedling$ or equine).ti,ab.

17. exp Dogs/ or cats/

18. (cat or cats or dog or dogs or feline or canine or ((small or companion) adj1 animal$)).ti,ab.

19. or/7-18

20. 3 and 6 and 19

21. limit 20 to english language

22. limit 21 to (comment or editorial or letter)

23. 21 not 22

24. remove duplicates from 23

Appendix 2. Search terms to return references in human populations and *in vitro* studies for screening in scoping review to identify modifiable factors or interventions to reduce the occurrence of antimicrobial resistance in cattle production systems.

1. exp Drug Resistance, Microbial/ or exp Drug Resistance, Bacterial/ or exp Drug Resistance, Multiple, Bacterial/
2. (resistanc$ or sensitivit$ or susceptib$).ti,ab.\
3. 1 or 2
4. expAcinetobacter/ or expBacteroides/ or expBordetella/ or expBurkholderia/ or expCitrobacter/ or exp Clostridium/ or expEnterobacteriaceae/ or exp Enterococcus/ or exp Escherichia coli/ or expEnterobacter/ or expHaemophilus/ or exp Helicobacter/ or expKlebsiella/ or exp Moraxella/ or exp Mycobacterium/ or exp Mycoplasma/ or exp Neisseria/ or exp Pseudomonas/ or exp Salmonella/ or expSerratia/ or expShigella/ or exp Staphylococcus/ or exp Streptococcus/ or expTreponema/ or exp Methicillin-Resistant Staphylococcus aureus/ or expVancomycin Resistance/ or exp beta-Lactamases/ or exp Tuberculosis/
5. (Acinetobacter$ or Bacteroides$ or Bordetella$ or Burkholderia$ or Citrobacter$ or Clostridi$ or Enterobacteriaceae$ or Enterococc$ or Escherichia coli$ or Enterobacter$ or Haemophilus$ or Helicobacter$ or Klebsiella$ or Moraxella$ or Mycobacterium$ or Mycoplasma$ or Neisseria$ or Pseudomonas$ or Salmonella$ or Serratia$ or Shigella$ or Staphylococcus$ or Streptococcus$ or Treponema$ or Methicillin Resistant Staphylococcus aureus or Vancomycin Resistance$ or betaLactamase$ or Tuberculosis$ or mrsa or vre or esbl).ti,ab.
6. 4 or 5
7. (alter$ or affect$ or effect$ or reduc$ or decreas$ or control$ or mitigat$ or interven$ or risk$ or factor$ or change$ or increas$ or modif$ of eliminat$).ti,ab.
8. exp Microbial Sensitivity Tests/
9. ((microbial or antibiotic$) adj2 sensitiv$).ti,ab.
10. exp Dose-Response Relationship, Drug/
11. pharmacodynamic$.ti,ab.
12. (dose adj2 response).ti,ab.
13. infection control/ or exp antisepsis/ or infection control, dental/ or patient isolation/ or quarantine/ or disinfection/
14. (infect$ adj3 control$).ti,ab.
15. (antibiotic adj4 manag$).ti,ab.
16. Cross Infection/
17. (cross adj1 infection$).ti,ab.
18. Or/8-17
19. 3 and 6 and 7 and 18

Limit 19 to English language

Appendix 3. Qualitative assessment instrument used to assess and classify animal-population studies screened during the scoping review to identify modifiable factors or interventions to reduce the occurrence of antimicrobial resistance in cattle production systems.

1. If an observational study, were the inclusion/exclusion criteria for the selection of the study population specified?

Choose one of the following answers.

1. Yes
2. Partially
3. Uncertain
4. No
5. NA

Please enter your comments here:

1. If a randomized or experimental type study design, were the groups appropriately defined and allocated?

Choose one of the following answers.

1. Yes
2. Partially
3. Uncertain
4. No
5. NA

Please enter your comment here:

1. For an experimental or randomized study design, could the absence of formal randomization influence the validity of the results?

Choose one of the following answers.

- 1. Yes
  2. Uncertain
  3. No
  4. NA

Please enter your comments here:

1. Were the procedures used in the study (e.g. questionnaires, data collection for risk factors, microbiology, antimicrobial susceptibility testing etc.) defined, appropriate and used correctly?

Choose one of the following answers.

- 1. Yes
  2. Partially
  3. Uncertain
  4. No

Please enter your comments here:

1. Were there biases that could have influenced the validity of the results?

Choose one of the following answers.

- 1. Yes
  2. Uncertain
  3. No
  4. NA

Please enter your comments here:

1. Were there confounders that were not adequately controlled that may have influenced the validity of the results?

Choose one of the following answers.

- 1. Yes
  2. Uncertain
  3. No
  4. NA

Please enter your comments here:

1. Were there problems with the statistical analysis that could have affected the validity of the results?

Choose one of the following answers.

- 1. Yes
  2. Uncertain
  3. No
  4. NA

Please enter your comments here:

1. Please provide an overall assessment of the quality of the data presented in this paper with respect to our objective.

Choose one of the following answers.

- 1. High
  2. Moderate
  3. Low
  4. Unreliable

Please enter your comments here:
